# Textile Dye Biodecolorization by Manganese Peroxidase: A Review

**DOI:** 10.3390/molecules26154403

**Published:** 2021-07-21

**Authors:** Yunkang Chang, Dandan Yang, Rui Li, Tao Wang, Yimin Zhu

**Affiliations:** 1 Institute of Environmental Remediation, Dalian Maritime University, Dalian 116026, China; cyk@dlmu.edu.cn; 2 The Lab of Biotechnology Development and Application, School of Biological Science, Jining Medical University, No. 669 Xueyuan Road, Donggang District, Rizhao 276800, China; yangdandan_jnmc@163.com (D.Y.); ruili061289@mail.jnmc.edu.cn (R.L.)

**Keywords:** manganese peroxidase, biodecolorization, dye wastewater, immobilization, recombinant enzyme

## Abstract

Wastewater emissions from textile factories cause serious environmental problems. Manganese peroxidase (MnP) is an oxidoreductase with ligninolytic activity and is a promising biocatalyst for the biodegradation of hazardous environmental contaminants, and especially for dye wastewater decolorization. This article first summarizes the origin, crystal structure, and catalytic cycle of MnP, and then reviews the recent literature on its application to dye wastewater decolorization. In addition, the application of new technologies such as enzyme immobilization and genetic engineering that could improve the stability, durability, adaptability, and operating costs of the enzyme are highlighted. Finally, we discuss and propose future strategies to improve the performance of MnP-assisted dye decolorization in industrial applications.

## 1. Introduction

The textile industry produces large quantities of wastewater containing different types of dyes used during the dyeing process, which cause great harm to the environment [1,2]. Many dyes and their intermediate metabolites have been identified as mutagenic, teratogenic, or carcinogenic, and represent serious health threats to living ecosystems [3].

At present, the treatment of dye wastewater mainly relies on physical or chemical management techniques, including chemical reduction, adsorption, ionizing radiation, precipitation, flocculation and flotation, membrane filtration, electric coagulation, electrochemical destruction, and ion exchange ozonation [4,5]. These technologies have obvious shortcomings such as the excessive use of chemicals, sludge production, expensive factory requirements or high operating expenses, low decolorization efficiencies, and the inability to handle large numbers of dyes with different structures, so they are not economically suitable for large-scale wastewater decolorization [6].

The current focus is to reduce toxicity and develop an efficient, economical, and green dye detoxification and decolorization technology. Compared with physical and chemical methods, biological methods offer beneficial and effective prospects due to their economical and environmentally friendly advantages, as well as being simple to use, safe, and efficient, with no secondary pollution [7,8]. Therefore, biotechnology is considered the best choice to degrade and remove these pollutants effectively. In the biotechnology field, enzyme biocatalysis is currently the main research area due to its broad application prospects [9,10].

Manganese peroxidases (EC 1.11.1.13; MnPs) are a family of heme-containing glycoproteins belonging to the oxidoreductase group. It was discovered in *Phanerochaete chrysosporium* and is also found in many bacteria and white-rot fungi (WRF) [11,12,13,14]. There are different MnPs in nature with differentiated properties. For example, long and short MnPs were reported in WRF associated with the presence/absence of the C-terminal tail extension, and these showed different catalytic and stability properties [15]. According to the residues of the Mn^2+^-binding site, three novel subfamilies of MnP were described in Agaricales including MnP-ESD (Glu/Ser/Asp Mn^2+^-oxidation site), MnP-DGD (Asp/Gly/Asp Mn^2+^-oxidation site), and MnP-DED (Asp/Glu/Asp Mn^2+^-oxidation site) [16]. However, the Mn^2+^-binding site is not the unique feature of MnPs, because versatile peroxidases (VPs), which evolved directly from MnPs, also possess such a site and can oxidize Mn^2+^ to Mn^3+^ [17].

For enzyme applications, MnPs can catalyze the peroxide-dependent degradation of a variety of toxic dye pollutants, phenolic compounds, antibiotics, and polycyclic aromatic hydrocarbons, so are promising biocatalysts for hazardous environmental contaminants biodegradation [18,19]. Moreover, the use of MnPs is suitable for dye wastewater decolorization as the process is simple and the enzyme can be recycled, thus reducing operating costs [20,21,22].

This review article summarizes the latest application of MnPs for wastewater dye decolorization and its future development prospects and challenges.

## 2. The Crystal Structure of MnPs

The crystal structure of an enzyme provides information on the catalytic mechanism and for potential in-depth design and transformation, and for realizing the green biotechnological use of enzymes [23,24,25].

The heme conformation of MnP is similar to that of lignin peroxidase (LiP) and is evolutionarily conserved [26]. In its resting-state form, MnP is a strongly helical protein containing a Fe^3+^ penta-coordinated structure with the porphyrin ring of the heme cofactor and a proximal histidine, with the sixth coordination position open for H_2_O_2_ [27].

**Figure 1:**
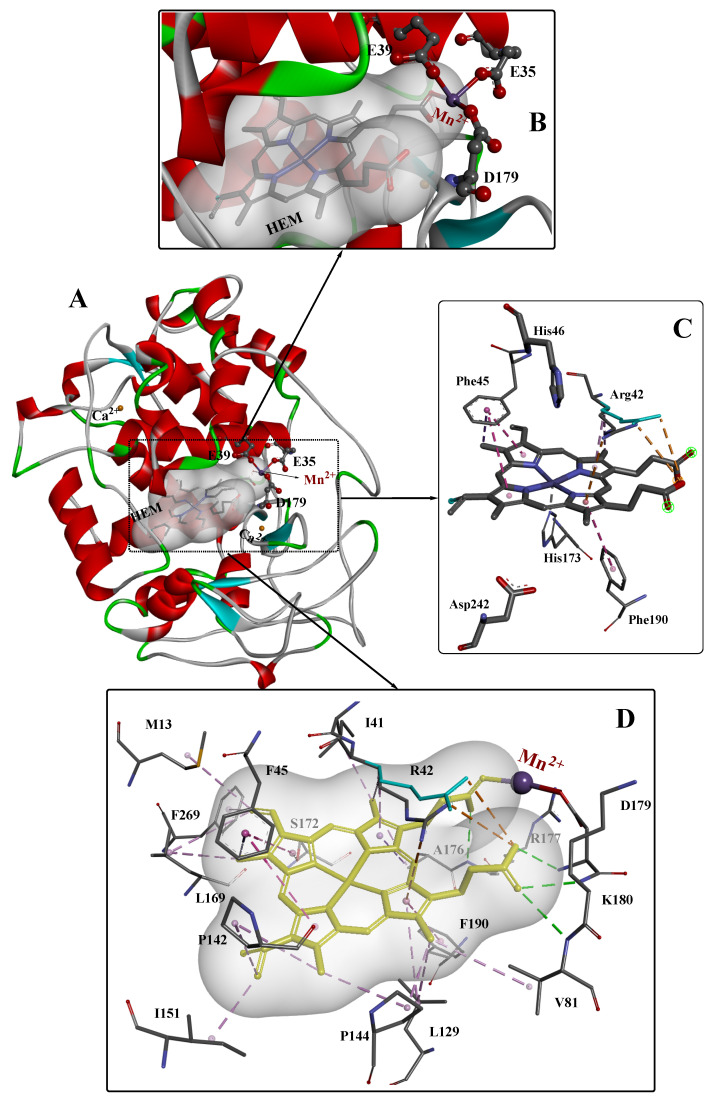
The overall structure (**A**), active site structure (**B**,**C**), and interaction mode (**D**) of Mn–MnP refined at 0.93 Å resolution [28]. PDB ID: 3M5Q.

To date, several crystal structures of MnP from different sources have been reported, and the highest-resolution crystal structures (~0.93Å) of MnP complexed with Mn^2+^ (Mn-MnP) are shown in Figure 1 [28]. The conserved Ca^2+^ ions are important for the stability of the protein [29]; these are indicated as gold yellow spheres and the position of the Mn^2+^ substrate is shown in violet. The active site is composed of three highly conserved amino acids (Glu35, Glu39, and Asp179) and one heme propionate. The Mn^2+^ substrate binds in the center of the active site, and the heme propionate (HEM) is located in the internal hydrophobic cavity of the enzyme. The spatial structure of HEM is further stabilized by four hydrogen bonds (green dashed line), two electrostatic interactions (orange dashed line), and some other weak interactions. The catalytic site of heme peroxidases is strongly conserved, with only minor variations occurring in the replacement of Phe with Trp in several enzymes such as ascorbate peroxidase and cytochrome c. The Asp–His pair (242 and 173, respectively) is also conserved.

## 3. MnP Catalysis

**Figure 2:**
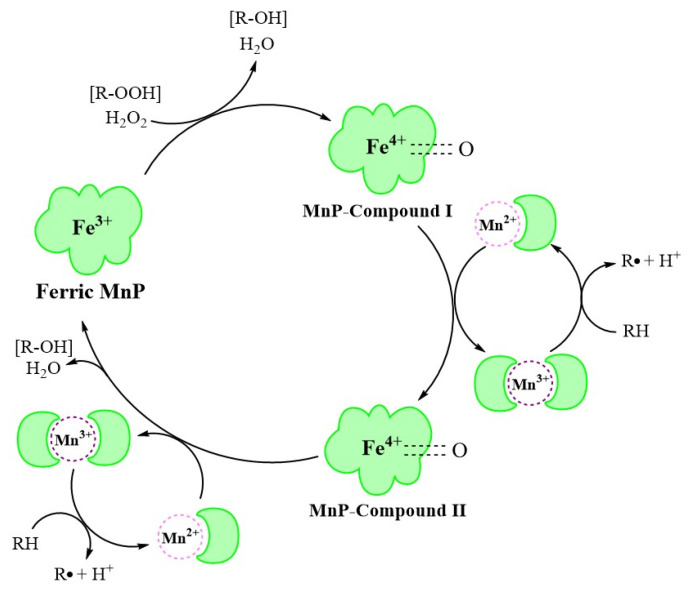
The MnP catalytic cycle [30].

At the beginning of the catalytic cycle, H_2_O_2_ or organic peroxide binds to the enzyme in resting state in ferric (Fe^3+^) form (Figure 2). This process releases one molecule of H_2_O and forms MnP–compound I (Fe^4+^-oxo-porphyrin radical complex), with two oxidation equivalents. This oxidizes Mn^2+^ to Mn^3+^, forming MnP–compound II (Fe^4+^-oxo-porphyrin complex). Immediately afterwards, the MnP–compound II combines with Mn^2+^ in a similar manner to generate Mn^3+^, releasing one molecule of H_2_O, and is reduced to the original state of ferric MnP, completing the catalytic cycle [30].

The MnP catalytic cycle resembles that of other lignin and heme peroxidases in the presence of native Fe^3+^ enzymes and two reactive intermediates [31]. However, in contrast to other peroxidases, MnP preferentially uses Mn^2+^ as the substrate, converting it to the strong oxidation state of Mn^3+^ through a series of redox reactions [32].

## 4. Application of Unmodified MnPs in the Decolorization of Dye Wastewater

Table 1 contains a summary of recent studies on the breakdown and decolorization of textile-derived dye compounds by microbial MnPs.

Singh and Dwivedi [33] reported the optimal conditions for decolorization of high concentrations of Direct Blue-1 (DB-1) by the fungus *Aspergillus terreus* GS28. This biodegradation was accomplished via an atypical symmetrical oxidative pathway using a combination of MnP and laccase, reducing the toxicity of the product. The maximum decolorization rate (98.4%) was recorded for an initial dye concentration of 100 mg/L within 168 h.

Generally, the decolorization rate is related to the type of dye, the initial dye concentration, media composition, experimental conditions, and the enzyme source. Rekik et al. [22] reported the purification of an MnP (named MnP TP55) from the recently isolated basidiomycete WRF fungus *Trametes pubescens* strain i8. The MnP TP55 was used to degrade different types of dyes including anthraquinone dyes (Remazol Brilliant Blue Reactif and Cibacet Brilliant Blue BG), triphenylmethane dye (Methyl Green), indigo dye (Indigo Carmine), azo dyes (Direct Red 5B and Remazol Brilliant Violet 5R), polymeric dye (Poly R-478), and acid dye (Acid Blue 158), reaching decolorization rates of 42%, 46%, 50%, 64%, 66%, 76%, 88%, and 95%, respectively, within 24 h at the initial dye concentration of 50 μM. Other similar research [34] reported that the purified extracellular MnP BA30 from *Bjerkandera adusta* strain CX-9 could degrade Acid Blue 158, Poly R-478, Cibacet Brilliant Blue BG, and Remazol Brilliant Violet 5R at rates of 91%, 80%, 77%, and 70%, respectively, within 12 h at the same initial dye concentrations of 50 μM.

In addition, the decolorization rate is positively related to the decolorization time, and the longer the decolorization time, the higher the decolorization rate obtained. Therefore, a high decolorization rate does not necessarily mean that the decolorization efficiency is equally high. Noman et al. [35] used various dyes (Remazol Brilliant Blue R (RBBR), Methylene Blue, and Congo Red) to investigate the decolorization abilities of fungal enzymes including Lip and MnP. The results revealed that the decolorization rate of RBBR ranged from 17.96 to 44.89% after 7 days, and between 55.98 and 99.99% after 15 days incubation.

Moreover, MnP is able not only to decolorize various dyes, but also to degrade other pollutants including polycyclic aromatic hydrocarbons (PAHs). Zhang et al. [36] purified an MnP from the WRF *Trametes* sp.48424 that was able to efficiently degrade various dyes. The results showed that the removal efficiencies of Indigo Carmine, RBBR, Remazol Brilliant Violet 5R, and Methyl Green were 94.6%, 85.0%, 88.4%, and 93.1%, respectively, within 18 h from initial dye concentrations of 100 mg/L. The enzyme was also demonstrated to have the ability to degrade PAHs in complex environments.

Most of the MnP-assisted decolorization in the above research made use of pure cultures, which are conducive to studying mechanisms; however, the effects are usually not as good as those of a microbial consortium. A fungal consortium, SR, comprising *Trametes* sp. SQ01 and *Chaetomium* sp. R01 was used for the decolorization of various triphenylmethane dyes [20] and was found to be more efficient than using the individual fungi. The consortium produced 1.3 U/mL of MnP, 5.5-fold more than that that generated by the *Trametes* monoculture. At the initial dye concentration of 100 mg/L, the decolorization rates of Coomassie Brilliant Blue G250 and Cresol Red were 96% and 93%, respectively, by the consortium within 6 days, significantly higher than those accomplished by *Trametes* alone (72% and 60%, respectively). Two further studies [37,38] reported the use of two microbial consortia named ZSY (mainly composed of Unclassified_f_Bacillaceae, *Piscibacillus*, and *Bacillus*) and ZW1 (mainly composed of *Halomonas*, *Marinobacter*, and *Clostridiisalibacter*), both of which reached more than 93% decolorization of 100 mg/L Metanil Yellow G within 48 h. In addition, both consortia were highly effective in high-pH and high-salinity environments, indicating potential use for the treatment of high-salinity and alkaline textile wastewaters.

## 5. Emerging Technology for the Use of MnPs in Decolorization

At present, the study of enzyme-assisted biodecolorization is developing rapidly, and many new technologies have been developed to improve decolorization efficiency and adaptability, and reduce the cost of treatment. Among them, the most representative new technologies are immobilization and the genetic engineering of enzymes [49].

### 5.1. Immobilization of MnPs

While enzymes are green, efficient, and widely used biocatalysts, the use of free enzymes is usually accompanied by limitations such as poor stability during storage and operation, high sensitivity to the environment, relative ease of deactivation, and difficulty in recycling, which represent major restrictions for their industrial application [50,51].

The immobilization of enzymes relies on carrier materials that restrict the enzyme to a specific location while retaining its catalytic activity and allowing recycling and reuse. Compared with free enzymes, immobilized enzyme technology has shown great advantages in improving both the stability and life of enzymes, and has become a research focus in recent years [52,53,54].

**Figure 3:**
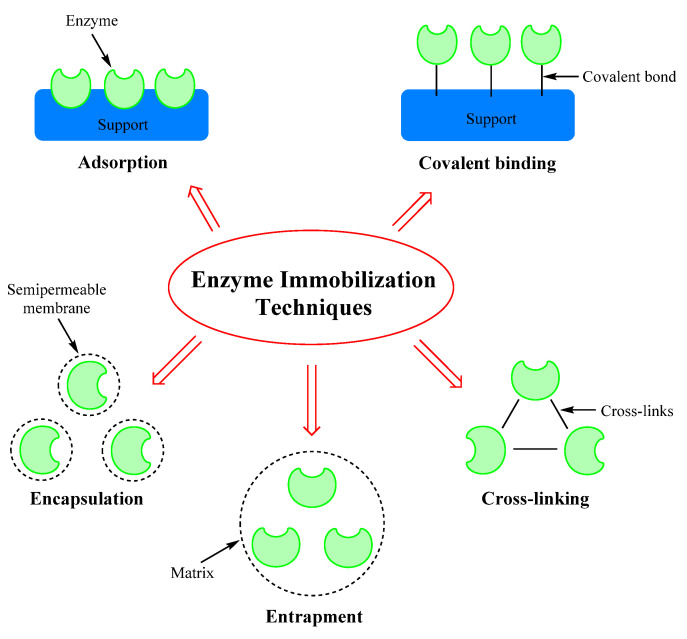
The most commonly used techniques for immobilization of enzymes [53].

Different MnP immobilization techniques have been applied for the degradation of toxic pollutants. These include physical methods such as entrapment and adsorption and chemical methods such as cross-linking and covalent bonding, illustrated in Figure 3 [53]. Table 2 lists the common methods of immobilizing enzymes and their advantages and disadvantages.

The activity of MnP tends to diminish during both storage and the enzymatic process, especially when hydrogen peroxide is present [64]. Therefore, MnP immobilization is a promising technology for its industrial application as it improves the enzyme’s resistance to changes in pH and temperature, as well as its stability during storage and use. In addition, the immobilized MnP is reusable, an advantage compared with free MnP.

Table 3 summarizes recent reports describing MnP immobilization in practical applications of dye decolorization, which have achieved results in enhancing its activity, stability, and durability.

Siddeeg et al. [65] immobilized *Anthracophyllum discolor* fungal MnP on magnetic nanocomposite Fe_3_O_4_/chitosan surfaces. The results showed that the immobilization of MnP increased its capacity to decolorize Methylene Blue and Reactive Orange 16, with removal efficiencies of 96 ± 2% and 98 ± 2%, respectively. The nanocomposite was also found to retain its activity after five successive cycles. Another study [21] immobilized MnP from *Ganoderma lucidum* IBL-05 on Ca-alginate beads. The results showed that the decolorization efficiency of five textile dyes by the immobilized MnP reached 82.1–95.7%, which was maintained after seven cycles. It is worth noting that the Ca-alginate-bound MnP retained high catalytic activity and operated over wide temperature and pH ranges, demonstrating promising industrial application prospects for dye removal from textile wastewater.

Moreover, immobilized MnP is not only effective for dye decolorization, but could also play a significant role in improving other indicators of water quality and reducing the toxicity of pollutants. Bilal et al. [66] described MnP immobilization by cross-linking with glutaraldehyde-activated chitosan beads. In investigating the detoxification and degradation of textile wastewater dyes, they found a maximum dye removal of 97.31% with up to 82.4%, 78.3%, and 91.7% reductions in chemical oxygen demand, total organic carbon, and biological oxygen demand, respectively. The cytotoxicity of the effluent after treatment was significantly reduced, and the root length, root number, and mitosis index of *Allium cepa* increased significantly, while the mortality of brine shrimp nauplii and the mutagenicity of the test strain decreased significantly. Moreover, the MnP retained 60% of its activity after 10 successive decolorization treatments. These results indicate the superior efficiency of immobilized MnP for treating textile effluents and suggest its use for remediating toxic material in wastewater.

In addition, enzyme immobilization technology does not necessarily immobilize only a single enzyme or use only one immobilization method; the co-immobilization of two or more cooperative enzymes or the combined use of multiple immobilization methods can often enhance the catalytic effect [67,68]. For example, one study [69] co-immobilized MnP and LiP on chitosan microspheres and tested the ability of these two nonspecific enzymes from *Coriolus versicolor* to cooperate in the breakdown of a variety of dye pollutants. The results showed that over 80% of the immobilized enzyme activity was retained after storage at 50 °C for 6 h, with 70% activity remaining after two weeks despite the free enzymes being inactive under the same conditions. Another study [70] reported the novel immobilization of MnP as cross-linked enzyme aggregate (CLEA) microcapsules and demonstrated the efficient decolorization of a dye mixture composed of nine different dyes. This showed a maximum decolorization (82%) of Brilliant Blue R (BBR) under optimal conditions, with percentages of MnP recycling efficiency after BBR exposure of 84%, 69%, 54%, and 40% after the second, third, fourth, and fifth cycles, respectively. Up to 36% of MnP activity remained even after 60 days’ storage.

In addition to the immobilization of free enzymes, several studies have directly immobilized microbial cells, which could also achieve satisfactory effects. One of these studies [71] demonstrated effective decolorization of Reactive Black 5 by *Pleurotus sajor-caju* immobilized onto a natural fiber of *Luffa cylindrica*, achieving decolorization rates of 75% within 10 h. More notably, MnP became more expressive when the microorganism was immobilized, with the increase of enzyme activity up to 41.55% on Day 10, thus demonstrating effective retention of the catalytic activity.

In summary, immobilized MnP shows outstanding catalytic, thermal, and storage stability, and this, together with its recyclability and potential for bioremediation, suggests its efficacy and potential for the treatment of textile industry effluents.

### 5.2. Genetic Engineering of MnPs

MnP has been widely researched due to its enzymatic properties and potential industrial applications. However, its widespread use has been mainly hindered by two main factors: limited enzyme production and low stability [81]. Different approaches have been explored to overcome these issues; for example, most research on yield improvement has focused on the optimization of the growth conditions and culture media composition. However, current research has demonstrated the effectiveness of genetic and strain engineering in improving both the yield and stability of the enzyme, and this has become a research hotspot in recent years [82,83]. In addition, both homologous and heterologous expression systems have been investigated to enhance MnP transcription, with heterologous expression achieved in various expression systems such as those based on bacteria and fungi [84,85,86].

**Figure 4:**
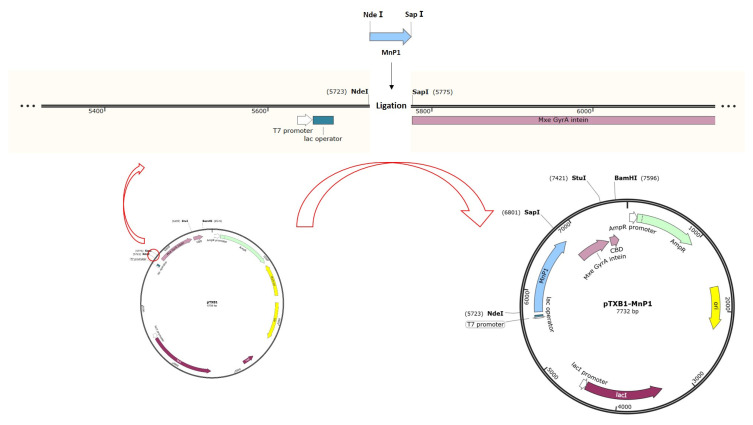
Schematic representation for the construction of the recombinant plasmid pTXB1-MnP1 [87].

For example, the synthetic MnP1 gene was cloned into the pTXB1 vector using *Nde*I/*Sap*I restriction sites and delivered into *Escherichia coli* DH5α cells [87]. The overall process for the construction of pTXB1-MnP1 is summarized in the diagram depicted in Figure 4. This research described a novel molecular approach for the expression and purification of MnP because of the recombinant MnP1 (rMnP1) was expressed as a fusion protein, which was recovered from solubilized inclusion bodies and could be purified from the fusion protein using intein-based protein purification techniques and a one-step affinity chromatography.

Lee et al. [88] reported the heterologous expression of a novel MnP gene from *Peniophora incarnata* KUC8836 in *Saccharomyces cerevisiae* and observed that this MnP was secreted more efficiently into the culture medium in this case than in wild-type *S. cerevisiae*. Xu et al. [89] cloned the *Ganoderma lucidum* MnP gene (*GluMnP1*) and transferred it into *Pichia pastoris* SMD116. The recombinant protein was expressed effectively, showing improved oxidation activity in the decolorization of four dye types as well as phenol degradation.

While recombinant MnP may not necessarily show improvement in its efficiency of decolorization, there may be significant enhancement of its stability or durability as well as in its ability to degrade other environmental pollutants such as polycyclic aromatic hydrocarbons. Chen et al. [90] cloned a novel MnP gene (*imnp*) from a new WRF, *Irpex lacteus* F17, successfully expressing it in *Escherichia coli* Rosetta (DE3). This recombinant MnP was stable over a wide pH range (from pH 3.5 to 9) and was able to decolorize azo dyes. Its maximum decolorization (80.2 ± 1.0%) of Reactive Black 5 was observed after 90 min at pH 3.5 and 35 °C. Zhang et al. [91] reported the cloning and expression of a novel MnP gene (*mnp3*) from WRF *Cerrena unicolor* BBP6 which, after expression in *Pichia pastoris*, showed an MnP activity of 154.5 U/L. The enzyme could decolorize various dyes and was also effective for bleaching denim. It was also able to degrade fluorene and phenanthrene, demonstrating its potential in both textile industry and polycyclic aromatic hydrocarbon bioremediation applications. Another similar study [92] reported the use of an *Aspergillus niger* strain to express an MnP gene (*mnp1*) from *Phanerochaete chrysosporium*, finding that the recombinant *A. niger* SBC2-T3 strain acquired MnP activity and could remove 95% of 400 ppm phenanthrene from a microcosm soil system within 17 days, in contrast to the wild type which removed 72% under the same conditions.

These genetic engineering findings indicate that recombinant MnP has significant potential for the bioremediation of persistent pollutants.

## 6. Conclusions and Prospects

Biodecolorization has gained prominence as an environmentally friendly, cheap, and effective technology for textile wastewater treatment [40,93,94]. This review shows the potential of MnP in a variety of biotechnological fields, especially in the treatment of dye wastewater. In addition, the development of enzyme immobilization and genetic engineering technologies has significantly improved the catalytic stability and capacity of biocatalysts as well as reducing processing costs. However, many challenges still limit its practical application in large-scale commercial applications.

Future research should concentrate on (1) the in-depth evaluation of the degradation mechanisms, operating parameters, and favorable conditions for microbial growth; (2) the creation of novel immobilization supports to reduce enzyme loss and improve enzyme durability; (3) the improvement of existing immobilization methods to protect the structure of the catalytic site and catalytic performance; (4) genetic engineering of microbial strains with enhanced biodegradation characteristics; and (5) further exploration of the feasibility of application of MnP-assisted decolorization in the large-scale treatment of actual dye wastewater.

## Figures and Tables

**Table 1:** Recent applications of unmodified MnPs in dye decolorization.

Source	Types of Dyes	Initial Concentration of Dyes	Removal Rate	Time Cost	Reference
Microbial consortium SR	Crystal Violet	20 mg/L	63%	6 days	[20]
	Cresol Red	100 mg/L	93%		
	CBB G250	100 mg/L	96%		
*Trametes pubescens* strain i8	Acid Blue 158	50 μM	95%	24 h	[22]
	Poly R-478		88%		
	Remazol Brilliant Violet 5R		76%		
	Direct Red 5B		66%		
	Indigo Carmine		64%		
	Methyl Green		50%		
	Cibacet Brilliant Blue BG		46%		
	Remazol Brilliant Blue Reactif		42%		
*Aspergillus terreus* GS28	Direct Blue-1	100 mg/L	98.4%	168 h	[33]
*Bjerkandera adusta* strain CX-9	Acid Blue 158	50 μM	91%	12 h	[34]
	Poly R-478		80%		
	Cibacet Brilliant Blue BG		77%		
	Remazol Brilliant Violet 5R		70%		
*Trametes* sp.48424	Indigo Carmine	100 mg/L	94.6%	18 h	[36]
	Remazol Brilliant Blue R		85.0%		
	Remazol Brilliant Violet 5R		88.4%		
	Methyl Green		93.1%		
Microbial consortium ZSY	Metanil Yellow G	100 mg/L	93.39%	48 h	[37]
Microbial Consortium ZW1	Methanil Yellow G	100 mg/L	93.3%	16 h	[38]
*Trichoderma harzianum*	Alizarin Blue Black B	0.03%	92.34%	14 days	[39]
*Phanerochaete chrysosporium* CDBB 686	Congo Red	50 ppm	41.84%	36 h	[40]
	Poly R-478		56.86%		
	Methyl Green		69.79%		
*Bjerkandera adusta* CCBAS 930	Alizarin Blue Black B	0.01%	86.5%	20 days	[41]
	Acid Blue 129		89.22%		
*Cerrena unicolor* BBP6	Congo Red	100 mg/L	53.9%	12 h	[42]
	Methyl Orange		77.6%	12 h	
	Remazol Brilliant Blue R		81.0%	5 h	
	Bromophenol Blue		62.2%	12 h	
	Crystal Violet		80.9%	12 h	
	Azure Blue		63.1%	24 h	
*Phanerochaete chrysosporium*	Indigo Carmine	30 mg/L	90.18%	6 h	[43]
*Trametes versicolor*	Dye mixture (Brilliant Blue FCF and Allura Red AC)	100 mg/L	80.45%	14 days	[44]
*Irpex lacteus*			86.04%	19 days	
*Bjerkandera adusta*			82.83%	9 days	
*Ceriporia lacerata* ZJSY	Congo Red	100 mg/L	90%	48 h	[45]
*Bacillus cohnni* RKS9	Congo Red	100 mg/L	99%	12 h	[46]
*Schizophyllum commune* IBL-06	Solar Brilliant Red 80	0.01%	100%	3 days	[47]
*Irpex lacteus* CD2	Remazol Brilliant Violet 5R	50 mg/L	92.8%	5 h	[48]
	Remazol Brilliant Blue R		87.1%	5 h	
	Indigo Carmine		91.5%	5 h	
	Direct Red 5B		82.4%	36 h	

**Table 2:** The various immobilization methods and their advantages and disadvantages [53,55,56,57,58,59,60,61,62,63].

Methods	Description	Advantages	Disadvantages
Adsorption	Free enzymes are adsorbed to the surface of the carrier through ionic bonds or van der Waals forces or hydrogen bonds.	Easy; Reusable; Low cost; Convenient and fast operation; No chemical changes.	Low efficiency; Nonspecific adsorption; Easily leaks from the carrier; Product contamination.
Entrapment	Free enzymes are embedded in the network structure formed by a gel.	Easy operation; High efficiency; Small damage to the enzyme; Good tolerance to extreme environments; High mechanical stability.	Large steric hindrance; Continuous enzyme leakage; Pore diffusion restraint; Not suitable for large scale or industrial process.
Encapsulation	Free enzymes are enclosed in selectively permeable membranes, such as nitrocellulose or nylon.	Cost effective; Durable; Easy preparation.	Mass transfer limitation; Unwanted byproducts; Enzyme leakage; High enzyme concentration required.
Cross-linking	Bifunctional or multifunctional cross-linking reagents are used to form covalent bonds between free enzyme molecules and cross-linking reagents.	Resistance to extraordinary pH and temperature circumstances; Increased rigidity of the structure; Durable; Good stability.	High cost; Difficult reaction control; Large enzyme quantity required; Loss of catalytic properties.
Covalent Binding	Free enzymes are linked to the carrier with inert groups or active groups by covalent bonding.	High efficiency; High thermal stability; Suitable for continuous reaction; No enzyme leakage.	High cost; Loss of catalytic properties.

**Table 3:** Recent applications of immobilized MnP in dye decolorization.

Carrier	Types of Immobilization	Results	Reference
Polyvinyl alcohol-alginate beads	Entrapment	High decolorization for different dyes (78.14–92.29%); Retained 64.9% enzyme activity after 6 cycles.	[21]
Magnetic nanocomposite Fe_3_O_4_/chitosan	Adsorption	96% and 98% decolorization of methylene blue and reactive orange 16, respectively; Reusable after 5 cycles.	[65]
Glutaraldehyde activated chitosan beads	Cross-linking Adsorption	Maximum decolorization reached 97.31%; 82.4%, 78.3%, and 91.7% reductions in COD, TOC, and BOD, respectively; Retained 60% enzyme activity after 10 cycles.	[66]
Glutaraldehyde activated chitosan microspheres	Cross-linking Adsorption	Retained over 80% enzyme activity at 50 °C for 6 h; Retained 70% enzyme activity after 2 months storage.	[69]
Glutaraldehyde Carboxymethyl cellulose-alginate beads	Cross-linking Encapsulation	Maximum decolorization reached 82%; Retained 69% and 40% activity after 3rd and 5th cycle, respectively; Retained 36% enzyme activity after 60 days storage.	[70]
Glutaraldehyde	Cross-linking	Maximum decolorization reached 100%.	[72]
Hydrophobic sol-gel comprising tetramethoxysilane and propyltrimethoxysilane	Entrapment	Maximum decolorization reached 100%; Retained 82% and 75% enzyme activity at pH 4 and 70 °C, respectively, after 24 h.	[73]
Ca-alginate	Entrapment	High decolorization for different dyes (82.1–95.7%); 94.85%, 95%% and 95.47% reductions in COD, TOC, and BOD, respectively; Reusable after 7 cycles.	[74]
Hydrophobic sol-gel matrix of tetramethoxysilane and propyltrimethoxysilane	Entrapment	Maximum decolorization reached 93.92%; Retained over 70% enzyme activity after 3 cycles.	[75]
Gelatin matrix using glutaraldehyde as cross-linking agent	Entrapment Cross-linking	Over 90% decolorization; Retained over 50% enzyme activity after 6th cycle.	[76]
Agar-agar support	Entrapment	High decolorization for different dyes (78.6–84.7%); Retained activity to 71.9% and 60.3% at 30 °C and 40 °C, respectively, after 120 h; Reusable after 10th cycle.	[77]
Agarose beads	Entrapment	Maximum decolorization reached 98.4%; Retained activity to 41.2% and 33.6% at 55 °C and 60 °C, respectively, after 72 h; Reusable after 10 cycles.	[78]
Xerogel matrix composed of trimethoxysilane and propyltetramethoxysilane	Entrapment	98.8% decolorization; Retained 82% enzyme activity after 10 cycles; Stored at 4 °C up to 75 days without significant activity loss.	[79]
Ca-alginate	Entrapment	Almost 90% decolorization; Retained 63% enzyme activity after 4 cycles.	[80]
